# Improved Classification Performance of Bacteria in Interference Using Raman and Fourier-Transform Infrared Spectroscopy Combined with Machine Learning

**DOI:** 10.3390/molecules29132966

**Published:** 2024-06-21

**Authors:** Pengjie Zhang, Jiwei Xu, Bin Du, Qianyu Yang, Bing Liu, Jianjie Xu, Zhaoyang Tong

**Affiliations:** State Key Laboratory of NBC Protection for Civilian, Beijing 102205, China; zpjbit@163.com (P.Z.); xujw14@mail.ustc.edu.cn (J.X.); dubin51979@163.com (B.D.); qyyang918@163.com (Q.Y.); lbfhyjy@sohu.com (B.L.); xujianjie@sklnbcpc.cn (J.X.)

**Keywords:** pollen interference, bacterial classification, feature fusion, machine learning, random forest

## Abstract

The rapid and sensitive detection of pathogenic and suspicious bioaerosols are essential for public health protection. The impact of pollen on the identification of bacterial species by Raman and Fourier-Transform Infrared (FTIR) spectra cannot be overlooked. The spectral features of the fourteen class samples were preprocessed and extracted by machine learning algorithms to serve as input data for training purposes. The two types of spectral data were classified using classification models. The partial least squares discriminant analysis (PLS-DA) model achieved classification accuracies of 78.57% and 92.85%, respectively. The Raman spectral data were accurately classified by the support vector machine (SVM) algorithm, with a 100% accuracy rate. The two spectra and their fusion data were correctly classified with 100% accuracy by the random forest (RF) algorithm. The spectral processed algorithms investigated provide an efficient method for eliminating the impact of pollen interference.

## 1. Introduction

The rise in questionable particulate matter in the air has resulted in a higher mortality rate. Rapidly detecting bacterial aerosols, especially as biological warfare agents, is crucial for human health protection [1,2,3]. The monitoring technology based on Raman spectroscopy and Fourier-Transform Infrared (FTIR) spectroscopy was mainly aimed at the future monitoring and warning needed of harmful biological aerosols and was divided into two directions: Raman Lidar detection and passive FTIR remote sensing. Raman Lidar actively emits lasers into the atmosphere and detects the distribution profile of aerosols by receiving atmospheric echo signals [4]. As a new field to be developed, it has not yet been used to identify biological aerosols. Although passive infrared remote sensing technology is affected by low instrument sensitivity and weak scattering or absorption of biological aerosols relative to background radiation, it could be used for detecting the concentration of biological aerosols and has the potential to develop into a new biological aerosol recognition technology [5]. The identification of biological warfare agents such as bacteria may be affected by other environmental substances, with pollen being the primary source of interference [6,7,8]. Therefore, reducing or eliminating the interference of pollen on bacterial classification and recognition was the focus of the research work.

The impact of the Raman and FTIR spectral characteristics of pollen on the recognition of bacterial categories cannot be ignored [9,10,11,12]. This is because pollen and bacteria both have Raman peaks on the amide I and amide III bands, protein C-H vibrations, phenylalanine characteristic peaks, and tyrosine circular respiratory vibrations [13,14]. The bands of amide I and amide II overlap, and there are multiple peaks of amino acids and polysaccharides in the FTIR spectrum. The spectral peaks of substances in the same category are very similar, and it is not easy to distinguish them using ordinary methods. Up to now, the underlying reasons for the impact of pollen spectral features on bacterial spectral feature classification and recognition have been unclear [15,16]. The continuous development of machine learning algorithms (MLAs) has brought new opportunities to research spectral classification and recognition technology [17,18,19,20]. We studied the relationship between the spectral features of pollen and other substances, used algorithms to extract spectral features, and successfully classified them.

With the development of pattern recognition theory, it is believed that there will be more significant improvements in signal processing technology in Lidar systems. At the same time, the addition of high-performance MLA also provided an excellent analytical tool for revealing the influence of pollen FTIR spectral features. With the increasing variety of biological warfare agents being monitored, it is meaningful to establish a more comprehensive feature spectrum database. A comprehensive spectral database laid the foundation for the future development of online monitoring technology. The collection method of aerosol particles has been used for laboratory static FTIR spectral classification research [21]. By combining this method with a classification model, spectral features were extracted and classified to identify unknown biological particles [22,23]. The classification performance of the model was related to spectral features, which improved as the spectral features of the input model increased. Simply adding spectral features could not continuously improve the model. The fusion and application of information from different spectra heralded a new approach to spectral feature analysis.

The development of new monitoring technology required models to have good classification performance for various biological particles. Three classes of bacteria were used to replace the original pathogenic bacteria in the laboratory to establish a bacterial detection model for both Raman and attenuated total reflectance Fourier-Transform Infrared (ATR-FTIR) spectra. The static Raman and ATR-FTIR spectra of all samples were collected and preprocessed. Subsequently, the spectral data were processed using feature extraction and classification algorithms. The classification ability of the model has been improved through the fusion of spectral features. This study attempted to explore methods for removing interference from pollen spectral features using machine learning. Raman spectral features were used to eliminate the interference between pollen and bacteria and classify them. In this study, the ATR-FTIR and Raman spectral features of pollen and bacteria were fused for the first time, weakening the influence of pollen by adding characteristic spectra. This strategy has great potential in mixed spectral classification and the recognition of targets under interference conditions. This lays a theoretical foundation for the development of real-time monitoring and warning devices for biological warfare agents based on these models in the future.

## 2. Results

### 2.1. Peak Assignments of Spectrum

**Figure 1:**
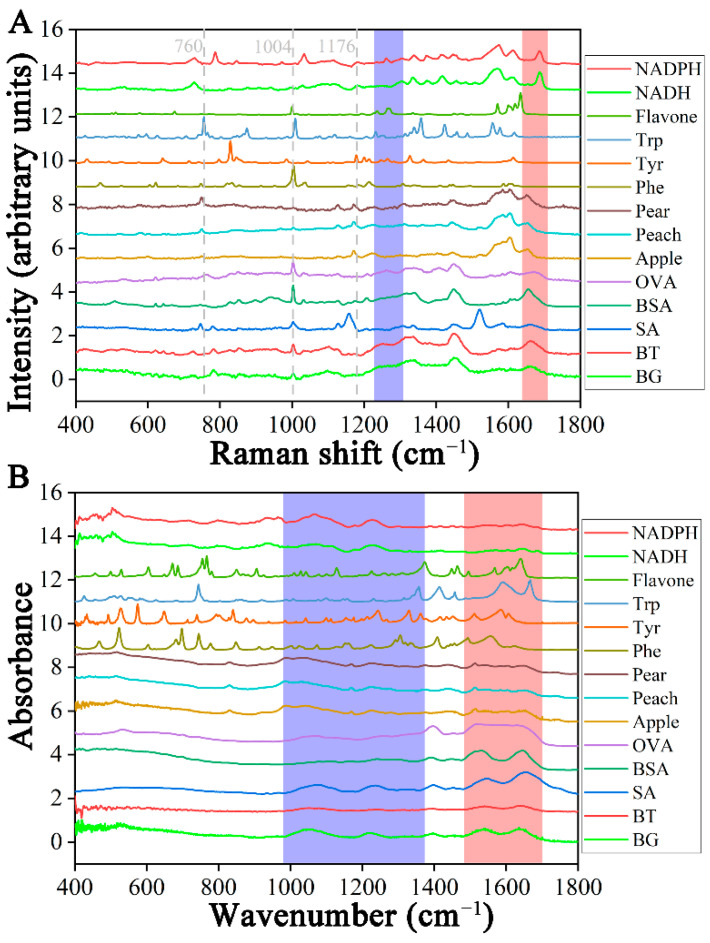
Raman and Fourier-Transform Infrared spectra and their characteristic peaks of fourteen samples. (**A**) Raman spectra. The shadow regions in each spectrum correspond to the amide III (purple) and amide I (orange) bands. (**B**) ATR-FTIR spectra. The shadow regions in each spectrum correspond to the lipid (purple) and protein (orange) bands.

The normalized fingerprint Raman spectra are shown in Figure 1A. The region between 400 and 1800 cm^−1^ was rich in molecular vibration information. Thus, some researchers used this region to identify biological samples [24,25]. The peak bands, such as amide I (1640–1680 cm^−1^), amide II (1480–1580 cm^−1^), amide III (1200–1300 cm^−1^), and disulfide bond (490–550 cm^−1^), were associated with proteins. The peak at 1209 cm^−1^ was the tryptophan and phenylalanine C-C_6_H_5_ vibration mode. The peak at 1004 cm^−1^ was a phenylalanine symmetric ring breathing. The ATR-FTIR spectra of the same region were normalized (Figure 1B). The peaks 980–1354 cm^−1^ and 1475–1710 cm^−1^ were the region of nucleic acids and amino acids, respectively. The peak at 1600–1700 cm^−1^ was an a-helices in amide I, and 1635 cm^−1^ was C=O stretching. The peak at 1458 cm^−1^ was the deformation vibration of C-H, and 1656 cm^−1^ was the stretching vibration of C=C and C=O, all related to protein [26]. The assignment of some peaks is displayed in Table 1.

The characteristic peak information of the Raman spectrum of the sample was more abundant than that of the ATR-FTIR spectrum, especially in the region at 1000–1800 cm^−1^, with the most significant differences between the various samples. Generally, protein-rich species have peaks in the red band. The reference samples showed significant differences in the 400–1800 cm^−1^ region compared to the ATR-FTIR of three bacterial targets. The spectral peaks mentioned may appear during the process of spectral feature extraction and classification.

### 2.2. Classification Models

#### 2.2.1. Principal Component Analysis

**Figure 2:**
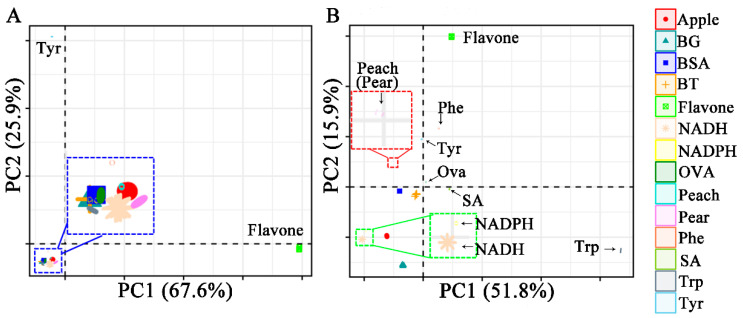
Principal component analysis between fourteen samples. (**A**) Raman spectra, (**B**) ATR-FTIR spectra.

The Raman and ATR-FTIR spectra were preprocessed by normalization, multiplicative scatter correction (MSC), and the Savitzky–Golay algorithm (SG) methods. The selection of the principal components (PCs) was crucial for extracting differences between different samples and aimed to explain the direction of the maximum variance of high-dimensional data. The principal component analysis (PCA) results showed Raman data clustering (Figure 2A). The PC1-PC2 scores plot of PCA modeling of flavone and amino acids showed apparent clustering. The three types of bacteria overlapped entirely, resulting in a classification failure. The points of proteins (Bovine serum albumin, BSA; Ovalbumin, OVA, Beijing, China) overlapped bacteria and were close to nicotinamide adenine dinucleotide phosphate (NADPH, Shanghai, China). From the results, it can be inferred that the composition of bacteria was similar to these proteins and amino acids. Peach overlapped with OVA, and pear pollen was closer to nicotinamide adenine dinucleotide (NADH, Shanghai, China).

Figure 2B shows the visualization results of the PCA analysis of ATR-FTIR. It can be observed that the samples follow specific trends between categories. *Bacillus atrophaeus* (BG), *Bacillus thuringiensis* (BT), and *Staphylococcus aureus* (SA, Beijing, China) are entirely separated. As a result of Raman spectrum analysis, BG was close to tryptophan (Trp, Shanghai, China), NADH, and apple pollen. The overlapping points of the two types of pollen can be understood as the slight difference in spectral characteristics between peach and pear pollen, resulting in similar PCA scores that were not being identified. The performance of PCA in classifying ATR-FTIR spectra seemed to be more prominent, with an accuracy of 85.7%. The ATR-FTIR spectroscopy can separate BG and BT, while Raman spectroscopy cannot. The ATR-FTIR spectroscopy did not separate peach from pear pollen, while Raman spectroscopy could. By combining the two spectra, all samples could be classified.

#### 2.2.2. Partial Least Squares Discriminant Analysis

**Figure 3:**
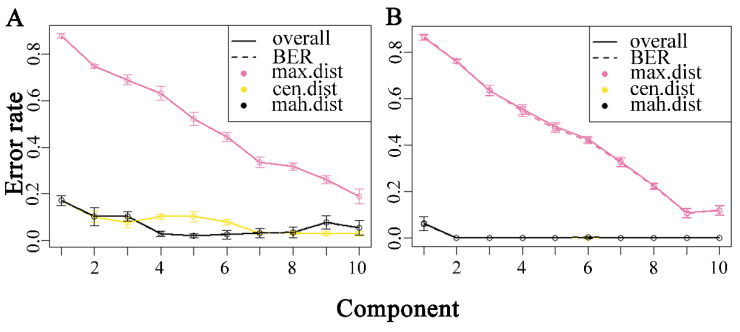
The relationship between the number of components and the variability in classification error rates observed in the partial least squares discriminant analysis model. (**A**) Raman spectra, (**B**) ATR-FTIR spectra.

For the development of a better bacterial identification model, partial least squares discriminant analysis (PLS-DA) was used to classify the unknown samples with the mixOmics package of R (version 4.3.1) on Raman and ATR-FTIR spectra. This model’s optimal number of components was selected based on the minimum balanced error rate (BER). The classification error rate was determined using five-fold random cross-validation, and the model performance was evaluated using the perf function in the R package, which was repeated ten times. As depicted in Figure 3A, the Mahalanobis distance (mah. dist) in Raman spectral data was at its minimum when the component number was 5. As the component number increased, the Mahalanobis distance initially rose and then declined. As shown in Figure 3B, the minimum Mahalanobis distance in FTIR spectral data was obtained at a component number of 2 and remained unchanged as the number increased.

**Figure 4:**
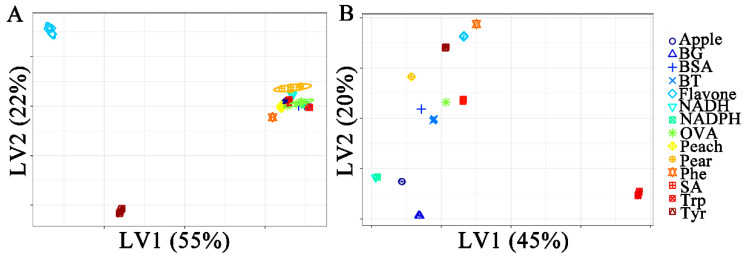
Partial least squares discriminant analysis between fourteen samples. (**A**) Raman spectra, (**B**) ATR-FTIR spectra.

Figure 4 shows the classification plots of Raman and ATR-FTIR spectra with two X-variates. In Figure 4A, the Raman spectra of flavonoids, Phenylalanine, and tyrosine are classified, and other samples overlap. As the optimal number of components increased, the separated sample species increased. In Figure 4B, most species are correctly separated in the PLS-DA model using the first two latent variables (LVs). However, two samples overlap in pairs: peach pollen and pear pollen. Two types of bacteria, BT and SA, were closer to BSA and OVA.

**Figure 5:**
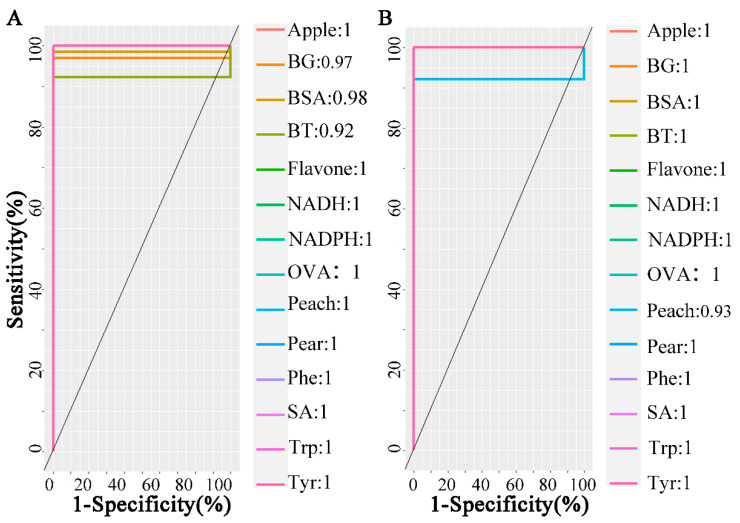
The receiver operating characteristic curve in partial least squares discriminant analysis model on samples. (**A**) Raman spectra, (**B**) ATR-FTIR spectra. In Raman data, the area under the receiver operating characteristic curve for three classes marginally deviates from the optimal value of 1. In Fourier-Transform Infrared spectra, the area under the receiver operating characteristic curve for peach pollen also falls below unity.

Furthermore, the cross-validation of the PLS-DA model showed that in Raman data, the area under the receiver operating characteristic curve (AUC) was relatively high, except for BG, BSA, and BT were slightly less than 1. However, in the ATR-FTIR data, all other samples were classified, except for peach blossom powder with AUC marginally less than 1, as shown in Figure 5A,B. The model achieved classification accuracies of 78.57% and 92.85%, respectively.

#### 2.2.3. Random Forest

**Figure 6:**
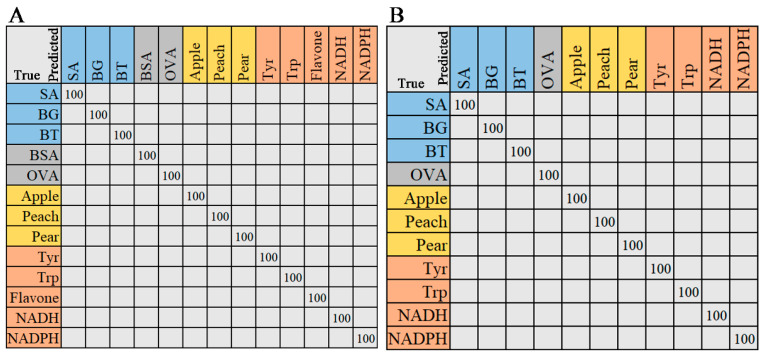
The confusion matrix of RF model. (**A**) Raman spectra, (**B**) ATR-FTIR spectra. The entries in the matrix represent the actual categories (rows) and the support vector machine-predicted categories (columns). The values on the diagonal line represent the precision of predictions for each category.

The random forest (RF) algorithm was employed for classification and regression analysis. The output of the RF classification model was the most-selected option, while the output of the regression model was the average of the results. As shown in Figure 6, the classification results of Raman and ATR-FTIR spectra are displayed in a confusion matrix. The ratio of the training and testing sets of the two data models was the same (7:3). The test set samples were correctly classified, with precision, recall, and F1-score values of 1. Both Raman and ATR-FTIR data demonstrate the excellent classification ability of RF. The processing time of the RF algorithm for Raman and IR spectra is 0.5612 s and 0.5910 s, respectively.

**Figure 7:**
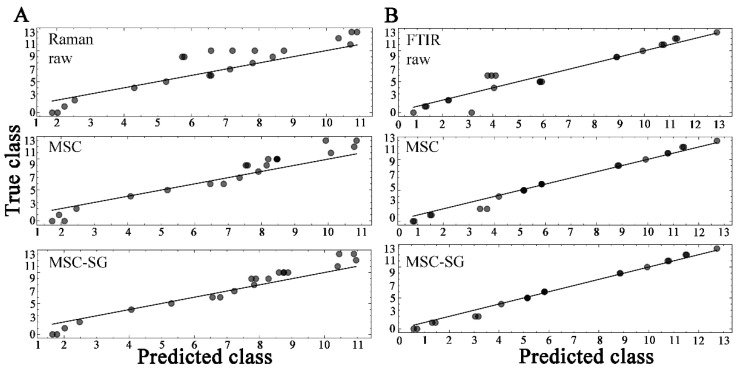
The regression results were derived from the RF model. (**A**) Raman spectra, (**B**) ATR-FTIR spectra. The spectrum is subjected to processing using three data processing methods. The more sample points on a straight line, the higher the accuracy of the model.

The sample labels were converted into numbers and used for regression analysis using RF. The labels of all samples are listed in Table S1, ranging from zero to thirteen. The prediction results of the RF model for Raman and ATR-FTIR spectra are shown in Figure 7. This strategy used raw data and two preprocessed data to test the performance of the RF model, and the processed data predicted better results. The data were randomly selected, so thirteen categories of Raman data were selected (no sample 3, i.e., Phe), while eleven categories of infrared data were selected (no sample 3,7,8, i.e., Phe, BSA, and flavone). Table 2 shows the results of different preprocessing models for spectral data, root mean square error of calibration (RMSEC) values, and the prediction of the validation set (RMSEP) as well as the coefficient of determination (R^2^) for the calibration ($R_{C}^{2}$) and prediction ($R_{P}^{2}$). Based on the prediction results in Table 2, the ATR-FTIR was better than the Raman spectra, and the data processed by MSC-SG were better than the original data. The ATR-FTIR data processed by MSC-SG had the best performance, with an RMSEP of 0.462, $R_{C}^{2}$ of 0.995, and $R_{P}^{2}$ of 0.988.

#### 2.2.4. Support Vector Machine

**Figure 8:**
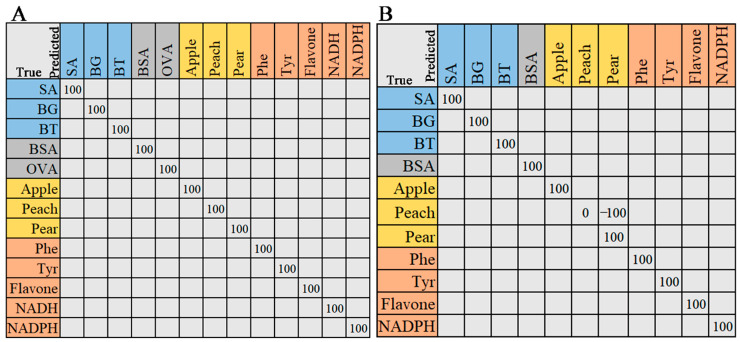
The confusion matrix was generated by the SVM model. (**A**) Raman spectra, (**B**) ATR-FTIR spectra. The entries in the matrix represent the true categories (rows) and the predicted categories (columns). The values on the diagonal line represent the precision of predictions for each category.

The support vector machine (SVM) was employed for the identification of various species in biological samples [33,34,35]. Here, we used fourteen samples with the same spectral range to demonstrate the feasibility of SVM. The data were divided into training and testing sets, with a ratio of 7:3. The classification results are shown in Figure 8. As shown in Figure 8A, the Raman samples were correctly classified, with precision, recall, and F1-score values of 1. The peach pollen in ATR-FTIR spectra was misclassified as pear, with a precision of 0. The precision of the pear was 0.33. The average accuracy (Average-Acc) was the mean value of each accuracy. The overall accuracy (Overall-Acc) was the ratio of the correct number of predictions to the total number of predicted samples. The root mean square error (RMSE) was the error between the predicted value and the actual value. The R^2^ of Raman and ATR-FTIR data had values of 1 and 0.9995, respectively, demonstrating a suitable fitting (Table 3). The processing time for the Raman spectrum and FTIT spectrum using the SVM algorithm was 1.4353 s and 0.7825 s, respectively.

#### 2.2.5. Classification Performance of Fused Spectral Features

**Figure 9:**
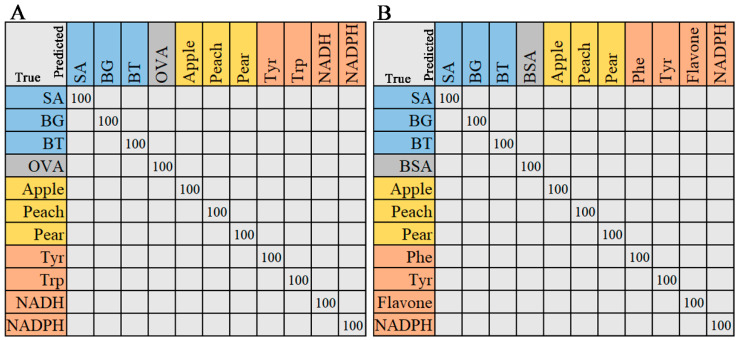
The confusion matrix illustrates the classification outcomes of the fused data obtained from Raman spectra and ATR-FTIR spectra. (**A**) RF, (**B**) SVM.

The FTIR spectral data were placed after the Raman spectral data, and a matrix of the two spectral data was then formed. The preprocessing of fusion spectral data remained consistent with the previous dataset. The classification performance of fusion spectral features was assessed using RF and SVM models. The fusion of Raman and ATR-FTIR spectra involved placing Raman features after FTIR features, increasing spectral features. Compared with a single spectrum, the RMSE of the fused spectra decreased, and the R^2^ value increased. As shown in Figure 9, the classification accuracy of the samples was 100%. The performance of fusion data is shown in Table 4. The results indicated that feature fusion is a new and effective way to improve the performance of spectral feature classification.

## 3. Discussion

This research has shown that analyzing their Raman and ATR-FTIR spectra can group bacteria based on their characteristics, using bacteria, bioactive substances, and pollen as samples. BG, BT, and OVA are common biological warfare agent stimulants. In addition to these two bacilli, a type of coccus, SA, was also added. This experiment considers that bacteria may be incomplete in real environments, with some proteins and amino acids exposed. Therefore, Bovine serum albumin and three amino acids were dropped into the sample pool. In addition, NADH and NADPH were important bioactive components involved in cell metabolism. Therefore, this study simulated the complex components of atmospheric aerosols, including the bacterial components and main interfering factors, as much as possible. At this point, a relatively simple and targeted combination of micro-atmospheric aerosols is established.

Raman and ATR-FTIR spectral features were used to identify the categories of substances. Because the infrared spectrum was easily affected by water, the sample to be tested is in a solid state. This study utilized the economic and rapid analysis of these dry powder substances, with sufficient reproducibility in the results. After collecting the spectral data information of all samples, multiple machine learning algorithms were used to conduct in-depth research on the modeling feasibility of each spectrum. The Raman signal was very weak, and, sometimes, it was not easy to obtain a good spectrogram. The confocal micro-Raman spectrometer enhanced the signal by nearly a hundred-times, with the advantages of high detection sensitivity, short time, low sample size, and no need for preparation. FTIR spectral data were collected using the attenuated total reflection module of the Nicolet iS50R spectrometer (Thermo Fisher Scientific Inc., Waltham, MA, USA), and there was no need to prepare samples. After simple baseline correction and smoothing processing by the instrument, the spectral data were saved as a comma-separated value (CSV) file. Then, the CSV format data were used for further analysis. Firstly, the data needed to be normalized. This was also a necessary step before starting machine learning algorithm training. Secondly, the data needed to go through preprocessing. The most important feature of data classification was consistency. Therefore, the correction of the baseline and the removal of noise elimination were important. The method of combining MSC and SG in this article met this requirement very well. Finally, the data needed to be selected and trained through classification algorithms. After training the spectral data, the model selected spectral features for sample classification.

Based on Raman and ATR-FTIR spectroscopy, the results indicated that bacteria and reference materials can be classified, their similarities can be identified, and the structural features of a few samples can be quickly analyzed using the described analysis methods. PCA and PLS-DA were used to classify bacteria based on the characteristics of spectra. The R^2^ refers to the sum of variance. The closer the value was to 1, the higher the quality of the model [36]. The RMSE, $R_{P}^{2}$, and $R_{C}^{2}$ were considered [37]. The accuracy, confusion matrix, and receiver operating characteristic curve were also used to evaluate the classification performance. The results showed that both PCA and PLS-DA can classify twelve different categories of samples through the spectral features of ATR-FTIR. The pollen and coenzymes were misclassified. However, both algorithms had poor classification performance but could separate pollen and samples by Raman spectral features. As a comparison, the Raman spectral features of pollen were more extracted by PCA and coenzymes by PLS-DA. The two spectra exhibited similar performance under the SVM model. SVM misclassified the ATR-FTIR of peach as pear. The classification accuracy of the RF algorithm for both spectra was 100%, indicating that the classification performance of RF was the best among these methods. Gao et al. constructed a fungal RF classifier to identify the geographic origin of Cabernet Sauvignon based on the composition of grape surface fungi, with an accuracy of 93.33% [38]. Lu et al. invented a method for classifying bacteria based on Confocal Micro-Raman Spectroscopy, with an average recognition rate of 97.21% [39]. Ramesh et al. provided classification results for only two types of bacteria based on the unique spectra of different pathogens [40]. The current scope of research on the classification, biological components, and pollen of bacteria was limited; thus, this study aimed to address this knowledge gap.

This work attempted to combine two spectral features to enhance the model’s recognition performance of bacteria under pollen interference. The SVM algorithm was employed to classify Raman spectra and ATR-FTIR spectral feature fusion data, demonstrating superior performance compared to FTIR data. The findings suggest that the integration of specific spectral features can effectively alleviate pollen interference, thus demonstrating the feasibility of combining multiple spectral techniques. The findings of these studies suggest that both Raman and ATR-FTIR spectra can be effectively employed for the classification of diverse biological samples, thereby emphasizing the significance of further research in this field. The present study established a theoretical research framework for the future advancement of multispectral detection technology. The practical application of this technology in the field presents a challenge that many researchers must consider.

## 4. Materials and Methods

### 4.1. Biological Samples

BG and BT were cultivated and provided by our laboratory. SA was purchased from the BeNa Culture Collection (Beijing, China) and cultivated in the laboratory. The absorbance (Abs) of bacterial suspensions during the logarithmic phase was measured. The Abs values for several bacteria were identical (value = 1.0). Bovine serum albumin (CAS:9048-46-8, 97%) and Ovalbumin (CAS:9006-59-1, biotechnology grade) were bought from Beijing Solarbio Science & Technology Co., Ltd. (Beijing, China). Three types of pollen powder (Apple, Peach, and Pear) were purchased from Taobao Online Mall. NADH (CAS: 606-68-8, coenzyme, 99%), Flavone (CAS: 525-82-6, 98%), Trp (CAS: 73-22-3, 99%), Tyrosine (CAS: 60-18-4, Tyr, 99%), and Phenylalanine (CAS: 63-91-2, Phe, 99%) were bought from Shanghai Aladdin Biochemical Technology Co., Ltd. (Shanghai, China). NADPH (CAS: 2646-71-1, coenzyme, 96%) was bought from Shanghai Macklin Biochemical Technology Co., Ltd. (Shanghai, China). Ultrapure water was used in the experiment (MILLIPORE, Billerica, MA, USA). Bacteria in the logarithmic phase were subjected to vacuum freeze-drying treatment, and the remaining reagents were used without further purification.

### 4.2. Attenuated Total Reflectance Fourier-Transform Infrared Spectral Measurements

The ATR-FTIR spectra were collected using a Nicolet iS50R FTIR spectrometer in the 400–3500 cm^−1^ range at 1 cm^−1^ resolution. A total of sixty-nine absorption spectra were recorded. A high-sensitivity DTGS detector was used for detection. The baselines of spectra were corrected using solution software.

### 4.3. Raman Spectral Measurements

Raman spectra were acquired with a resolution of 4 cm^−1^ in the 200–3500 cm^−1^ region using a DXR3 Raman Microscope (Thermo Fisher Scientific Inc., Waltham, MA, USA). All spectra were recorded under the same conditions: laser wavelength of 532 nm, laser power of 5 mW, and integration time of 6 s. A total of seventy Raman spectra (fourteen species (five replicates) were obtained. A blank background was documented after every five scans. The instrument software corrected the baselines of spectra (OMINIC Spectra 2.2.0, Waltham, MA, USA).

### 4.4. Data Treatment

The selected spectra preprocessing methods included spectral standardization, scattering correction, and smoothing, which are written in Python language. Z-score standardization was employed to standardize spectral data. This process included subtracting the original data from their mean, dividing by the standard deviation, and scaling the data to achieve a standard normal distribution with a mean of 0 and a variance of 1. The formula is as follows.

(1) $$x_{std}=\left(x_{i}-\mu \right)/\sigma$$

where *x_std_* was the standardized data; *x_i_* was the original data; *μ* was the mean data; *σ* was the standard deviation.

MSC was used to reduce the spectral noise. The mean value of the spectrum was taken as the ideal spectrum. Unitary linear regression was then performed between each spectral sample and the mean spectrum, solving the least square problem to obtain the regression constant *b* and regression coefficient *k*. The obtained *b_i_* and *k_i_* were used to correct the original spectra. The formula is as follows.

(2) $$A_{i\left(MSC\right)}=\left(A_{i}-b_{i}\right)/k_{i}$$

where *A_i_*_(*MSC*)_ was the output of MSC method; *A_i_* was the original data.

SG smoothing involved selecting a subset of the measured raw data as the window, with the smoothing window width being an odd 2*m* + 1. The measurement point was denoted as y, and the data within the window were fitted by a polynomial order *k*. The formulas are as follows.

(3) $$y\left(i\right)=a_{0}+a_{1}i+a_{2}i^{2}+\cdots +a_{k}i^{k}\;=\sum_{n=0}^{k}a_{n}i^{n},\;\;\left|i\right|\leq m$$

(4) $$\frac{\partial }{\partial a_{n}}=\left[\sum_{i=-m}^{m}{\left(y\left(i\right)-x_{i}\right)}^{2}\right]$$

(5) $$y_{i}=\sum_{j=-m}^{m}a_{j}^{\left(0\right)}x_{i+j}$$

where *a_n_* was polynomial coefficient; *x_i_* was the original signal; *y_i_* was the output of SG filter. Finally, the spectra were treated with multiplicative scatter correction MSC and the Savitzky–Golay algorithm SG to reduce the noise level.

The PCA, PLS-DA, SVM, and RF algorithms were applied to extract spectral features for the classification of biological components. The sample prediction was completed using fingerprint spectra (400–1800 cm^−1^) of Raman and infrared spectra.

### 4.5. Performance Evaluation Metrics

The model’s performance was evaluated by employing the R^2^ and RMSE. The confusion matrix (CM) accurately depicts the predictive results of the classifier. True positive (TP, positive samples correctly classified), false negative (FN, negative samples incorrectly classified), false positive (FP, positive samples incorrectly classified), and true negative (TN, negative samples correctly classified) were employed to evaluate the performance of the classifier.

The accuracy metric represents the ratio of accurate predictions to the total number of predictions made, as in Equation (6).

(6) $$\mathrm{Accuracy}=\frac{\mathrm{TP}+\mathrm{TN}}{\mathrm{TP}+\mathrm{TN}+\mathrm{FP}+\mathrm{FN}}$$

The precision is determined by dividing the count of accurately predicted positive instances by the total count of predicted positive class values, as in Equation (7).

(7) $$\mathrm{Precision}=\frac{\mathrm{TP}}{\mathrm{TP}+\mathrm{FP}}$$

The recall is computed as the ratio of true-positive predictions to the total number of actual positive values in the test dataset, as in Equation (8).

(8) $$\mathrm{Recall}=\frac{\mathrm{TP}}{\mathrm{TP}+\mathrm{FN}}$$

The F1-score represents the harmonic mean of precision and recall ratios, as in Equation (9).

(9) $$F1-\mathrm{score}=\frac{2*\mathrm{Precision}*\mathrm{Recall}}{\mathrm{Precision}+\mathrm{Recall}}$$

## 5. Conclusions

The Raman and ATR-FTIR spectra in this study were subjected to preprocessing techniques, including normalization, MSC, and SG smoothing methods. PCA, PLS-DA, RF, and SVM algorithms were employed for feature extraction and the classification of bacterial, pollen, and other biological samples. The classification performance of FTIR spectra surpassed that of Raman spectra when employing PCA and PLS-DA models. The classification performance of Raman spectra surpassed that of FTIR spectra when employing SVM. The random forest model demonstrated a classification accuracy of 100% for both data categories, underscoring its exceptional performance in accurately categorizing the data. The fusion features of Raman and FTIR spectra were effectively classified using RF and SVM models. The fusion spectral data exhibited superior classification performance compared to the FTIR spectrum in the SVM model. The constructed model effectively categorized fourteen types of biological sample spectra while efficiently mitigating the interference caused by pollen. The aforementioned methods lay the foundations for future advancements in online monitoring technology utilizing Raman and FTIR spectroscopy.

## Figures and Tables

**Table 1:** Raman and ATR-FTIR spectral feature assignments.

Wavenumber (cm^−1^)	Assignment	Reference
Raman spectroscopy		
1655–1680	Amide I (proteins), C=O stretching (lipids)	[27]
1300–1230	Amide III	[27]
1209	Tryptophan and phenylalanine ν(C–C_6_H_5_) mode	[27,28]
1176	C–H bending tyrosine (proteins)	[27]
1004	Phenylalanine	[27]
760	Ring breathing tryptophan (proteins)	[27]
ATR-FTIR spectroscopy		
1710–1475	Amino acids and proteins	[29]
1700–1600	Amide I	[30]
1600–1500	Amide II	[31]
1354–980	Lipids, nucleic acids, and carbohydrate	[32]

**Table 2:** Spectral classification performance of random forest regression model. Each spectrum is preprocessed using three methods, namely raw, multiplicative scatter correction, and multiplicative scatter correction—Savitzky–Golay. The root mean square error of calibration and validation and the correlation coefficient for the calibration and prediction are displayed.

Spectra	RMSEC	RMSEP	$R_{C}^{2}$	$R_{P}^{2}$
Raman				
raw	0.743	1.816	0.965	0.792
MSC	0.751	1.402	0.965	0.876
MSC-SG	0.694	1.200	0.970	0.909
ATR-FTIR				
raw	0.388	1.141	0.990	0.925
MSC	0.322	0.619	0.993	0.978
MSC-SG	0.270	0.462	0.995	0.988

**Table 3:** Performance of support vector machine classification model. The root mean square error of calibration and the correlation coefficient are shown. The overall accuracy is the ratio of the correct number of predictions to the total number of predicted samples.

Spectra	Average-Acc	RMSE	R^2^	Overall-Acc
Raman	1	0	1	1
ATR-FTIR	0.9090	0.3085	0.9995	0.9048

**Table 4:** The performance evaluation of random forest and support vector machine models for processing fused data. The Raman and ATR-FTIR features are concatenated to form the fused data. The root means square error of calibration and validation, and the correlation coefficient for the calibration and prediction are shown.

Spectra	Average-acc	RMSE	R^2^	Overall-Acc
RF	1	0	1	1
SVM	1	0	1	1

## Data Availability

The data presented in this study are available in article and Supplementary Materials.

## Supplementary Materials

The following supporting information can be downloaded at: https://www.mdpi.com/article/10.3390/molecules29132966/s1, Table S1: The number and abbreviation of fourteen samples.

## References

[1] Lei Y., Tian Z., Sun H., Zhu Z., Liang W., Li A. (2021). Self-cleaning and flexible filters based on aminopyridine conjugated microporous polymers nanotubes for bacteria sterilization and efficient PM_2.5_ capture. Sci. Total Environ..

[2] Li K., Yang Y., Xu C., Ye Y., Huang L., Sun L., Cai Y., Zhou W., Ge Y., Li Y. (2023). Vertical gold nanowires-based surface-enhanced Raman scattering for direct detection of ocular bacteria. Sensor Actuators B Chem..

[3] Romano S., Di Salvo M., Rispoli G., Alifano P., Perrone M.R., Tala A. (2019). Airborne bacteria in the Central Mediterranean: Structure and role of meteorology and air mass transport. Sci. Total Environ..

[4] Li S., Di H., Li Y., Yuan Y., Hua D., Wang L., Chen D. (2021). Detection of aerosol mass concentration profiles using single-wavelength Raman Lidar within the planetary boundary layer. J. Quant. Spectrosc. Radiat. Transf..

[5] Schütze C., Lau S., Reiche N., Sauer U., Borsdorf H., Dietrich P. (2013). Ground-based Remote Sensing with Open-path Fourier- transform Infrared (OP-FTIR) Spectroscopy for Large-scale Monitoring of Greenhouse Gases. Energy Procedia.

[6] Chen Q., Li J., Hua X., Jiang X., Mu Z., Wang M., Wang J., Shan M., Yang X., Fan X. (2020). Identification of species and sources of atmospheric chromophores by fluorescence excitation-emission matrix with parallel factor analysis. Sci. Total Environ..

[7] Gopalakrishnan S., Arigela R., Thyagarajan S., Raghunathan R. (2022). Comparison and evaluation of enumeration methods for measurement of fungal spore emission. J. Aerosol Sci..

[8] Sengupta A., Laucks M.L., Dildine N., Drapala E., Davis E.J. (2005). Bioaerosol characterization by surface-enhanced Raman spectroscopy (SERS). J. Aerosol Sci..

[9] Ben-David A., Ren H. (2003). Detection, identification, and estimation of biological aerosols and vapors with a Fourier-transform infrared spectrometer. Appl. Opt..

[10] Doughty D.C., Hill S.C. (2017). Automated aerosol Raman spectrometer for semi-continuous sampling of atmospheric aerosol. J. Quant. Spectrosc. Radiat. Transf..

[11] Gong Z., Pan Y.L., Videen G., Wang C. (2018). Optical trapping-Raman spectroscopy (OT-RS) with embedded microscopy imaging for concurrent characterization and monitoring of physical and chemical properties of single particles. Anal. Chim. Acta.

[12] Tripathi A., Jabbour R.E., Guicheteau J.A., Christesen S.D., Emge D.K., Fountain A.W., Bottiger J.R., Emmons E.D., Snyder A.P. (2009). Bioaerosol analysis with Raman chemical imaging microspectroscopy. Anal. Chem..

[13] McKenna O.E., Posselt G., Briza P., Lackner P., Schmitt A.O., Gadermaier G., Wessler S., Ferreira F. (2017). Multi-Approach Analysis for the Identification of Proteases within Birch Pollen. Int. J. Mol. Sci..

[14] Patel S., Rani A., Goyal A. (2017). Insights into the immune manipulation mechanisms of pollen allergens by protein domain profiling. Comput. Biol. Chem..

[15] Gottardini E., Rossi S., Cristofolini F., Benedetti L. (2007). Use of Fourier transform infrared (FT-IR) spectroscopy as a tool for pollen identification. Aerobiologia.

[16] Jin H., Wang J., Jin S., Jiang L., Zou Y. (2020). Raman spectroscopy of potential bio-hazards commonly found in bio-aerosols. Spectrochim. Acta A Mol. Biomol. Spectrosc..

[17] Feng C., Zhao N., Yin G., Gan T., Yang R., Chen X., Chen M., Duan J. (2021). Artificial neural networks combined multi-wavelength transmission spectrum feature extraction for sensitive identification of waterborne bacteria. Spectrochim. Acta A Mol. Biomol. Spectrosc..

[18] Maya-Manzano J.M., Tummon F., Abt R., Allan N., Bunderson L., Clot B., Crouzy B., Daunys G., Erb S., Gonzalez-Alonso M. (2023). Towards European automatic bioaerosol monitoring: Comparison of 9 automatic pollen observational instruments with classic Hirst-type traps. Sci. Total Environ..

[19] Nabatchian A., Abdel-Raheem E., Ahmadi M. (2011). Illumination invariant feature extraction and mutual-information-based local matching for face recognition under illumination variation and occlusion. Pattern Recognit..

[20] Wang Y., Ni H., Li H., Chen J., Zhang D., Fu L. (2022). Plasmonic microneedle arrays for rapid extraction, SERS detection, and inactivation of bacteria. Chem. Eng. J..

[21] Frain M., Schmidt D.P., Pan Y.-L., Chang R.K. (2006). Selective Deflection and Localization of Flowing Aerosols onto a Substrate. Aerosol Sci. Technol..

[22] Lu W., Li H., Qiu H., Wang L., Feng J., Fu Y.V. (2023). Identification of pathogens and detection of antibiotic susceptibility at single-cell resolution by Raman spectroscopy combined with machine learning. Front. Microbiol..

[23] Tang J.-W., Li J.-Q., Yin X.-C., Xu W.-W., Pan Y.-C., Liu Q.-H., Gu B., Zhang X., Wang L. (2022). Rapid Discrimination of Clinically Important Pathogens through Machine Learning Analysis of Surface Enhanced Raman Spectra. Front. Microbiol..

[24] Dikec J., Pacheco M., Dujourdy L., Sandt C., Winckler P., Perrier-Cornet J.M. (2023). Influence of hydration on calcium dipicolinate (CaDPA) during UVb and UVc exposure studied via Raman, FTIR and O-PTIR spectroscopy. J. Photochem. Photobiol. A Chem..

[25] Sun J., Xu X., Feng S., Zhang H., Xu L., Jiang H., Sun B., Meng Y., Chen W. (2023). Rapid identification of salmonella serovars by using Raman spectroscopy and machine learning algorithm. Talanta.

[26] Moros J., Garrigues S., de la Guardia M. (2007). Evaluation of nutritional parameters in infant formulas and powdered milk by Raman spectroscopy. Anal. Chim. Acta.

[27] Notingher I., Green C., Dyer C., Perkins E., Hopkins N., Lindsay C., Hench L.L. (2004). Discrimination between ricin and sulphur mustard toxicity in vitro using Raman spectroscopy. J. R. Soc. Interface.

[28] Stone N., Kendall C., Smith J., Crow P., Barr H. (2004). Raman spectroscopy for identification of epithelial cancers. Faraday Discuss..

[29] Ferreira I.C.C., Aguiar E.M.G., Silva A.T.F., Santos L.L.D., Cardoso-Sousa L., Araujo T.G., Santos D.W., Goulart L.R., Sabino-Silva R., Maia Y.C.P. (2020). Attenuated Total Reflection-Fourier Transform Infrared (ATR-FTIR) Spectroscopy Analysis of Saliva for Breast Cancer Diagnosis. J. Oncol..

[30] Guleken Z., Unubol B., Bilici R., Saribal D., Toraman S., Gunduz O., Erdem Kuruca S. (2020). Investigation of the discrimination and characterization of blood serum structure in patients with opioid use disorder using IR spectroscopy and PCA-LDA analysis. J. Pharm. Biomed. Anal..

[31] Sheng D., Wu Y., Wang X., Huang D., Chen X., Liu X. (2013). Comparison of serum from gastric cancer patients and from healthy persons using FTIR spectroscopy. Spectrochim. Acta A Mol. Biomol. Spectrosc..

[32] Dou J., Dawuti W., Li J., Zhao H., Zhou R., Zhou J., Lin R., Lu G. (2023). Rapid detection of serological biomarkers in gallbladder carcinoma using fourier transform infrared spectroscopy combined with machine learning. Talanta.

[33] Sahu S.K., Pandey M. (2023). An optimal hybrid multiclass SVM for plant leaf disease detection using spatial Fuzzy C-Means model. Expert Syst. Appl..

[34] Thirumala K., Pal S., Jain T., Umarikar A.C. (2019). A classification method for multiple power quality disturbances using EWT based adaptive filtering and multiclass SVM. Neurocomputing.

[35] Wu S., Jia D.K., Liu X.L., Yan F.G., Li Y.F. (2011). Application of continuous wavelet features and multi-class sphere SVM to chatter prediction. Adv. Mater. Res..

[36] Galvin-King P., Haughey S.A., Elliott C.T. (2021). Garlic adulteration detection using NIR and FTIR spectroscopy and chemometrics. J. Food Compos. Anal..

[37] Leng T., Li F., Xiong L., Xiong Q., Zhu M., Chen Y. (2020). Quantitative detection of binary and ternary adulteration of minced beef meat with pork and duck meat by NIR combined with chemometrics. Food Control.

[38] Gao F., Zeng G., Wang B., Xiao J., Zhang L., Cheng W., Wang H., Li H., Shi X. (2021). Discrimination of the geographic origins and varieties of wine grapes using high-throughput sequencing assisted by a random forest model. LWT-Food Sci. Technol..

[39] Lu X., Huang Q., Miller W.G., Aston D.E., Xu J., Xue F., Zhang H., Rasco B.A., Wang S., Konkel M.E. (2012). Comprehensive detection and discrimination of Campylobacter species by use of confocal micro-Raman spectroscopy and multilocus sequence typing. J. Clin. Microbiol..

[40] Ramesh G., Paul W., Valparambil Puthanveetil V., Raja K., Embekkat Kaviyil J. (2022). Raman spectroscopy as a novel technique for the identification of pathogens in a clinical microbiology laboratory. Spectrosc. Lett..

