# Supplementary material for: Improved Classification Performance of Bacteria in Interference Using Raman and Fourier-Transform Infrared Spectroscopy Combined with Machine Learning
Source: Molecules. 2024 Jun 21;29(13):2966. doi: 10.3390/molecules29132966 (PMC11242951; doi:10.3390/molecules29132966)
Supplement: Supplementary file 1 [file molecules-29-02966-s001.zip › molecules-3046670-supplementary.pdf]

Improved the Classification Performance of Bacteria in Inter-ference using Raman and Fourier Transform Infrared Spec-troscopy Combined with Machine Learning

Pengjie Zhang [1], Jiwei Xu [1], Bin Du [1], Qianyu Yang [1], Bing Liu [1], Jianjie Xu [1] and Zhaoyang Tong [1,*]

State Key Laboratory of NBC Protection for Civilian, Beijing, 102205, China

*Corresponding author: Zhaoyang Tong, State Key Laboratory of NBC Protection for Civilian, Beijing, 102205, China

E-mail: billzytong@126.com

Table S1. The number and abbreviation of fourteen samples.

Number	Classes
0	Apple
1	Bacillus atrophaeus (BG)
2	Bacillus thuringiensis (BT)
3	Phenylalanine (Phe)
4	Tyrosine (Tyr)
5	Peach
6	Pear
7	BSA
8	Flavone
9	Tryptophan (Trp)
10	NADH
11	NADPH
12	OVA
13	Staphylococcus aureus (SA)
